# Pollution Distribution of Potentially Toxic Elements in a Karstic River Affected by Manganese Mining in Changyang, Western Hubei, Central China

**DOI:** 10.3390/ijerph18041870

**Published:** 2021-02-15

**Authors:** Zhao Liu, Ye Kuang, Shengtao Lan, Wenjia Cao, Ziqi Yan, Li Chen, Qianlong Chen, Qi Feng, Hong Zhou

**Affiliations:** Institute of Geological Survey, China University of Geosciences, Wuhan 430074, China; cugliuzhao@foxmail.com (Z.L.); 1201921041@cug.edu.cn (Y.K.); lanshengtao@foxmail.com (S.L.); 1201921039@cug.edu.cn (W.C.); 20141004202@cug.edu.cn (Z.Y.); lizi@cug.edu.cn (L.C.); 20131001096@cug.edu.cn (Q.C.); 20151002621@cug.edu.cn (Q.F.)

**Keywords:** potentially toxic element, manganese mining activity, pollution distribution, potential ecological risk

## Abstract

This study investigated the distribution, pollution level and potential ecological risk of potentially toxic elements (PTEs) from manganese mining in a karstic Danshui River, in Changyang, Western Hubei, Central China. River water and sediments were collected for seven PTEs measurement (As, Cd, Cr, Cu, Mn, Pb and Zn), as well as pH and Eh of the river water were measured. Results showed that the major pollutant was Mn, the river water environment was mainly acidic and oxidizing (288 < Eh, pH < 6.3), and the pollution distribution of Mn in the study area was dominated by the combination of natural processes and anthropogenic activities. In the river water, according to the contamination factor (CF) and pollution load index (I_PL_) results, Mn was considered the main pollutant. There was low As and Pb pollution downstream as well as Cu pollution upstream. Upstream and downstream areas were the main polluted river sections of the river water samples collected. In river sediments, based on the results of the geo-accumulation index (I_geo_) and potential ecological risk index (I_PER_), it was determined that there was only considerable Mn pollution. The I_PER_ of the PTEs from the river sediments was at acceptable levels, only Mn upstream performed at a moderate ecological risk level. According to Pearson correlation and principal component analysis, Mn originated from manganese mining activities, Cd, Cr and Zn were of natural origin, and Cu may have come from both mining and natural origin, whereas Pb and As were mainly related to the daily activities. Consequently, elemental speciation, mining activities and the distribution of water conservancy facilities were the main impacts of PET pollution distribution in this river.

## 1. Introduction

Potentially toxic elements (PTEs) are persistent pollutants with potential hazards [1,2,3]. They cannot be biodegraded and easily accumulate in water bodies, sediments and biosphere, which can be harmful to human health and the ecosystem [4,5,6,7]. However, they rarely accumulate to harmful levels within natural conditions [8,9]. PET pollution caused by anthropogenic activities, especially mining activities, are widely distributed in many countries all over the world [10]. The water environment (especially the important physical and chemical parameters: pH and Eh) is easily changed by mining activities, and then affects the migration and transformation of pollution elements in the water environment [11]. Numerous studies indicated that mining was a major source of PTEs [12,13] and rivers in the mining areas were the main medium to transport the pollutants. It has become increasingly important to distinguish between the geogenic and anthropogenic sources of these PTEs and to understand that the background values change depending on the area and the scale of the area investigated [14]. However, there were still few studies focused on the pollution levels of different sections of the river in mining areas [15,16,17]. Therefore, studies focused on the pollution levels along the rivers in mining areas are discussed below, which are of great significance to prevent the environmental pollution of rivers.

The Changyang Gucheng Manganese Mine is one of the eight major manganese mines in China, with an estimated reserve of about 37 million tons of manganese. It is located at the midstream of the Danshui River and it has been running by separation floating and electrolytic processes for more than 20 years [18,19]. According to the field investigations, its acid sewage and slag were discharged directly into the Danshui River in the early years and caused considerable pollution to the river water and sediments. Although the local manganese mine owners built tailings ponds above the Cretaceous aquiclude to store the acid sewage and slag, the acid sewage and slag can still leak during transportation. In addition, the manganese mine is still running today, which may continue to produce pollution. PTEs in the river slag and sediments from the manganese mining activities could also be released into the water again when the water environmental conditions change [20,21]. These PTEs pose a significant threat to the local water security, but there are limited studies on the pollution of these manganese mining activities.

As, Cd, Cr, Cu, Mn, Pb and Zn are typical PTE pollutants in manganese mining areas [22,23,24,25]. To assess the potential impacts of manganese mining on river water and sediments, the concentration of these seven toxic heavy metal elements in both river water samples and sediments from the Danshui River was collected and analyzed. In this paper, (1) the concentration values were compared to the Chinese surface water environmental quality standards III (GB3838-2002) [26] and the average concentration of PTEs in water samples and sediments from the uninfluenced areas upstream of the Danshui River; (2) statistical analysis tools such as Pearson Correlation and Principal Component Analysis (PCA) were used to identify the relationships between the PTEs and possible common sources of pollution; (3) different pollution and risk indices were calculated to evaluate the level of contamination and the possible ecological risks in different sections of the river.

## 2. Materials and Methods

### 2.1. Study Area

**Figure 1:**
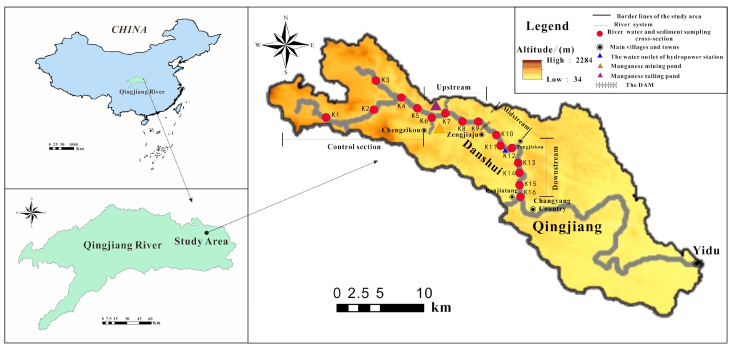
Location of the study area and sampling sites water samples and sediments of Danshui River.

The study area is located in the middle section of the Danshui River in Changyang County, southwest Hubei (Figure 1). This section is located in a karstic area. From the perspective of the geological background, the strata exposed in the study area are Cambrian and Ordovician carbonate strata consisting of mainly dolomite and limestone. The river sediments of the river were mainly silt, sand and carbonate gravel that mainly came from the local rock strata. From the perspective of the water system structure, the Danshui River is the primary tributary of the north bank in the lower Qingjiang River and branches from northwest to southeast. There is a large curve in the lower reach of the Danshui River and many water conservancy facilities are distributed along the river. The Gucheng manganese mine and its tailings pond are located on the left bank and the right bank, respectively, of the middle reach in Danshui. According to the hydraulic characteristics, the emission of pollutants and the distribution of the water conservancy facilities along the river can be divided into four sections (Figure 1): ① Control section: the section of the river not polluted by human activities [19]. ② The upstream of the study area: the main pollution discharge area of the manganese mining activities and there is a dam underneath (Chengzikou~Zengjiaju). ③ The midstream of study area: Zengjiaju~Pengjiakou. ④ The downstream of the study area: the acid sewage from upstream is discharged in the upper part of this section after being utilized by the hydropower station and there is a wasteyard in the lower part of this section (Pengjiakou~Panjiatang). In order to implement the protection plan of the Yangtze river from 2019, the local government adopted powerful measures to control the local pollution problems, which included improving the treatment process of the acid sewage and slag from manganese mining activities and building manganese tailings ponds and dams. However, due to the considerable pollution problems remaining from the earlier mining activities, it was necessary to assess the pollution status of PTEs in the river water and sediments.

### 2.2. Sample Collection and Chemical Analysis

Based on the Technical Code for Surface Water and Sewage Monitoring (HJ/T 91-2002) and the Code for Geochemical Reconnaissance (DZ/T 0011-2005) [27,28], 16 sampling cross-sections positioned along the Danshui River were identified (Control section: K1~K5, Upstream: K6~K9, Midstream: K10~K12, Downstream: K13~K16). A total of 32 samples (16 river water (RW) and 16 river sediments (RD)) were collected during October 2019. River water samples were collected at a depth of up to 30 cm and stored in 500 mL polyethylene bottles after filtering with a microfiltration membrane (pore size 0.45 μm) and acidified using 3 mg·L^−1^ of concentrated nitric acid [HNO_3_ (mass fraction: 98%)] in the field. The sample bottles were washed three times with the water sample to be collected prior to sampling. River sediments samples of about 1~2 kg were collected from 0~10 cm by a hand auger made of 304 stainless steel (YKD-20, Changsha Yonglekang Company, Changsha, China) and stored in clean zip lock polyethylene bags. The hand auger was cleaned three times with deionized water before each new sample was collected to avoid cross-contamination. Both river water and sediments samples were kept below 4 °C until further analysis.

All samples were measured within 14 days after sampling. The total concentrations of As, Cd, Cr, Cu, Mn, Pb and Zn were analyzed using inductively coupled plasma-optical emission spectrometry (ICP-OES, ICAP7600, ThermoFisher, Wuhan, China). The detection limit for each metal was 0.1 μg·L^−1^. In the experiment, the sample and blank control (i.e., adding HNO_3_ and ultra-pure water in the same proportion as the measured sample) were measured 6 times and the average value of the last three measurements was taken as the analysis result. After the sample was analyzed, the standard sample was measured again to ensure that the analysis error of data was controlled within 3%. After the sediment samples were air-dried, grounded, and sieved through 200 mesh screens, approximately a 0.1 g sediment sample was digested by HF–HNO_3_–HClO_4_ in a Teflon container and to a final liquid volume of 100 mL. Afterward, As, Cd, Cr, Cu, Mn, Pb and Zn in the sediment samples were quantitatively determined by mass spectrometry (ME-MS61, Agilent 7900, Guangzhou, China), and the detection limits of As, Cd, Cr, Cu, Mn, Pb and Zn were 0.002, 0.0002, 0.01, 0.002, 0.05, 0.005 and 0.02 mg·kg^−1^, respectively. The blank sample, parallel sample and national sediment standard sample GSD-12(GBW07312) were used for the quality control of the samples. The metal content of the blank samples was required to be less than 0.1 μg/L, the standard deviation between parallel samples less than 5%, and the error between the test value and the actual value of the standard sample less than 5% in order to pass quality control measures. The pH and Eh of the river water samples were measured in the field using a portable pH meter (Orion 8102BNUWP, Thermo Scientific, Waltham, MA, America) and acidity meter (PHJ-4A, Shanghai Leici Company, Shanghai, China), respectively. All physical parameters in river water samples were measured in triplicate and averaged.

### 2.3. Assessment of Pollution in River Water

#### 2.3.1. Contamination Factor (CF)

The contamination factor (CF) is an index for pollution status and is a widely used quantitative index to assess pollution levels in a given environmental compartment [29], which is calculated as:

(1) $$\mathrm{CF}=\frac{C_{s}}{C_{\mathrm{BG}}}$$

where C_s_ is the concentration of PTEs in a water sample (mg·L^−1^); C_BG_ is the background values of elements and was based on the river water concentration of PTEs in the control section (C_BG_ of As = 0.005, Cd = 0.002, Cr = 0.002, Cu = 0.01, Mn = 0.006, Pb = 0.008, Zn = 0.005 mg·L^−1^). Four levels were defined: low contamination (CF < 1); moderate contamination (1≤ CF < 3); severe contamination (3 ≤ CF < 6) and extreme contamination (CF ≥ 6) [29].

#### 2.3.2. Pollution Load Index (I_PL_)

In order to further evaluate the comprehensive status of the PTEs in a particular site or area in the river surface water samples. The pollution load index (I_PL_) was used based on the contamination factor (CF) [30], which followed the equation:

(2) $$I_{\mathrm{PL}}=\sqrt{{\mathrm{CF}}_{1}\times {\mathrm{CF}}_{2}\times \ldots \times {\mathrm{CF}}_{n}}$$

where n represents the number of PTEs. The grading criteria of the pollution load index are classified as: non-contaminated (0 ≤ I_PL_ < 1); baseline (I_PL_ = 1); progressive contamination (I_PL_ > 1) [31].

### 2.4. Assessment of Pollution in River Sediments

#### 2.4.1. Geo-Accumulation Index (I_geo_)

Geo-accumulation index (I_geo_) is a widely used method to evaluate pollution of PTEs in sediments [32,33]. Its formula is:

(3) $$I_{\mathrm{geo}}=\log_{2}(\frac{Y_{i}}{A\cdot B_{i}})$$

where Y_i_ is the concentration of potentially toxic element in the sediment (mg·kg^−1^), and B_i_ is the geochemical background concentration of the PTEs in the river sediment (the B_i_ of As = 19, Cd = 1, Cr = 82, Cu = 39, Mn = 487, Pb = 15, Zn = 85 mg·kg^−1^); A is the calibration factor and generally with 1.5; I_geo_ has seven grades (Table 1), interpreting as: I_geo_ ≤ 0—uncontaminated; 0 < I_geo_ ≤ 1—uncontaminated to moderately contaminated; 1 < I_geo_ ≤ 2—moderately contaminated; 2 < I_geo_ ≤ 3—moderately to heavily contaminated; 3 < I_geo_ ≤ 4—heavily contaminated; 4 < I_geo_ ≤ 5—heavily to extremely contaminated; I_geo_ ≥ 5—extremely contaminated [32,33,34].

#### 2.4.2. Potential Ecological Risk Index (I_PER_)

To assess the potential ecological risk of PTEs in river sediments, the Hakanson’s potential ecological risk (I_PER_) is used, which indicates the toxicology of PTEs [35,36,37]. The equation is:

(4) $$E_{r}^{i}=T_{r}^{i}\frac{C_{s}^{i}}{C_{n}^{i}}$$

(5) $$\mathrm{RI}=\sum_{i=1}^{7}E_{r}^{i}$$

where RI is the sum of potential ecological risks of all PTEs; $E_{r}^{i}$ is the potential ecological risk index of an individual potentially toxic element; $T_{r}^{i}$ is the biological toxicity factor of a given PET (the $T_{r}^{i}$ of As = 10, Cd = 30, Cr = 2, Cu = Pb = 5, Mn = Zn = 1) [38,39]; $C_{s}^{i}$ is the actual concentration of PTEs in river sediments (mg·kg^−1^); $C_{n}^{i}$ is the background concentration of PTEs in river sediments, which is consistent with B_i_. However, the classification of potential ecological risk index is based on 8 pollutants (As, Cd, Cr, Cu, Hg, Pb, PCB (Polychlorinated Biphenyl), Zn). The amount and type of PTEs may affect the assessment standards; thus, the grading criteria of ecological risk are improved (Table 1) [40,41].

## 3. Results and Discussion

### 3.1. Concentration Distribution of PTEs

**Figure 2:**
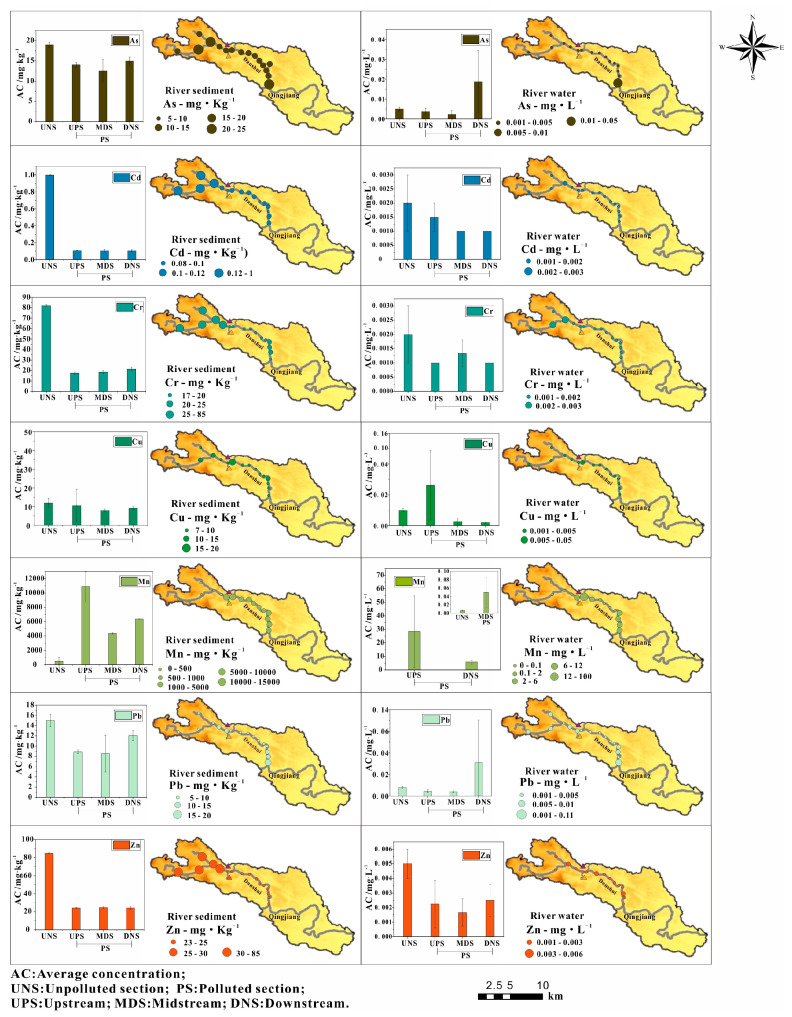
Distribution of PTEs in river water and sediments along the Danshui River in manganese mining area.

**Figure 3:**
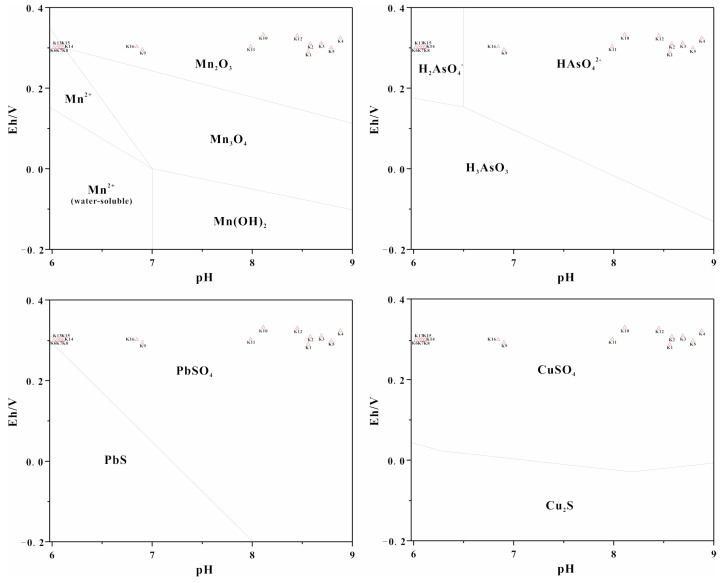
The stability fields of authigenic minerals as function of pH-Eh at 25 °C (modified from references [45,46,47,48]).

The coefficient of variation (CV) and concentration of PTEs in river water and sediments along the Danshui River are showed in Table 2 and Figure 2. The CV can intuitively show the spatial distribution characteristics of PTEs. The high variability (100% < CV) suggests that a significantly uneven spatial distribution, the medium variability (10% ≤ CV ≤ 100%) show a relatively homogeneous spatial distribution, and the low variability (CV < 10%) indicate a relatively even spatial distribution [42,43,44]. After the analysis, it was found that the concentration of Mn was 0.03~92.8 mg·L^−1^ and 3010~14150 mg·kg^−1^ in river water samples and sediments, respectively. The CV for the river water samples and sediments were 103 ± 2% and 206 ± 12%, respectively. These results indicate that the distribution of Mn in the water and sediments of the Danshui River was uneven [42,43,44]. The difference in the Mn of the river water and sediments was obvious, and was concentrated upstream and downstream relative to the mining operation with the average concentrations of 28.4 mg·L^−1^ and 5.9 mg·L^−1^ in waters. These values were 284 and 59 times higher than the GBIII levels, and 4733 and 983 times higher than the C_BG_ levels. The average concentration of Mn in the water of the midstream was lower than the GBIII, but it was 8.3 times higher than the C_BG_. Mn in the sediments from upstream to downstream were 10,915 mg·kg^−1^, 4367 mg·kg^−1^ and 6380 mg·kg^−1^, which were 22 times, 9 times and 13 times higher than the B_i_, respectively. According to literature research and field investigations, the over-standard phenomenon and spatial distribution of Mn in the study area were dominated by the combination of natural processes and anthropogenic activities. The Mn in the electrolytic manganese residue mainly existed in the form of oxides and the water environment at the sampling cross-sections with high Mn concentration in the study area (K6–K8 in upstream, K13–K15 in downstream) was mainly acidic and oxidizing conditions (295 < Eh, pH < 6.3, Table 3, Figure 3). Mn in river water existed in the valence state of Mn^2+^ and easily migrated in this environment, but the water environment at the other cross-sections with relatively low Mn concentration in river water (K1–K5 in the control section, K9 in lower upstream, K10–K12 in midstream, K16 in lower downstream) was mainly at moderately alkaline and oxidizing conditions (288 < Eh, 6.9 < pH, Table 3, Figure 3) where Mn existed in the form of Mn_2_O_3_, which is relatively stable [45,46,47]. In addition, based on the field investigation, PET pollutants were released mainly from upstream sources. Although a dam that sits between upstream and midstream ideally blocks the pollutants, the acid sewage from upstream was discharged to a downstream location after being utilized by the hydropower station. Thus, it is possible that the Mn from the river water samples and sediments mainly originated from the manganese mining activities. The excessive degree of Mn in the upstream and downstream of the study area was relatively high, while the midstream was lower affected and the control section formed a good contrast.

The concentrations of Pb and As in the river water were 0.002~0.1 mg·L^−1^ and 0.001~0.07 mg·L^−1^, and their CV were 35 ± 2% and 36 ± 3%, respectively. Similarly, the concentrations of Pb and As in the river sediments were 7.6~18.1 mg·kg^−1^ and 11.8~19.6 mg·kg^−1^, respectively, and their CV were 37 ± 2% and 35 ± 2%, respectively. It suggested that the distributions of Pb and As in river water and sediments were relatively uneven [42,43,44]. The concentration of Pb in the river water at the K16 location downstream (0.1 mg·L^−1^) was higher than the GBIII and C_BG_ levels. As distributed similarly to Pb, but it did not exceed the GBIII in the river. This metal was also high at the K16 sampling location with a concentration of 0.07 mg·L^−1^, which was 14 times higher than the C_BG_. In addition, Pb and As in the sediments were both high at the K16 sampling location with 18.1 mg·Kg^−1^ and 19.6 mg·Kg^−1^, which was 1.2 times and 1.03 times higher than the B_i_. These results suggest that As and Pb in the river water and sediments at K16 was highly affected by human activities. In addition, the water environment at the sampling cross-sections with high As and Pb concentrations in the study area (K6 in downstream) was mainly at alkaline and oxidizing conditions (288 < Eh, 8 < pH, Table 3, Figure 3). As and Pb in the river water existed in the valence state of As(V) (H_2_AsO_4_^−^ and HAsO_4_^2−^) and PbSO_4_ and were stable in the river water in this environment [45,46,47]. As and Pb concentrations in the other cross-sections in the river water were relatively uniform and irregular and their speciation had little influence on their spatial distribution. According to the field investigations, the K16 sampling location was near a wasteyard and it was inferred that the high concentrations of As and Pb in the river water and sediments at K16 were related to this facility. As and Pb at other sites along the river were all lower than the GBIII levels and their distributions were relatively uniform and irregular, thus they could be a result of natural origins [43].

Cu in the river water were 0.001~0.1 mg·L^−1^ and lower than the GBIII levels. Cu in the river sediments were 7.3 to 19.3 mg·Kg^−1^ and also lower than the B_i_. However, the CV of Cu in river water and sediments was 36 ± 3% and 55 ± 4%, respectively, which were uneven [42,43,44]. The average concentration of Cu in the water at the K7 sampling location which was upstream was higher than the C_BG_ levels, which was 0.1 mg·L^−1^. Although Cu in sediments at K7 with 19.1 mg·Kg^−1^ was lower than the B_i_, it was higher than other sites. However, the other sampling cross-sections did not exceed the standard, and the K7 was located in the main pollution discharge area of manganese mining activities. In addition, the Cu mainly exists in the form of oxides in the karst strata, and Cu existed in the state of CuSO_4_ in the river water of the study area and always easily migrated in acid and oxidizing environment (295 < Eh, pH < 6.3 Table 3, Figure 3) [12,46,47]. Therefore, Cu in river water and surface sediments at K7 may be related to manganese mining activities. However, Cu in river water and sediments at the other cross-sections were relatively uniform and irregular, so it was determined that Cu at the other cross-sections also originated from nature [43].

Cd, Cr and Zn in the river water were 0.001~0.002 mg·L^−1^, 0.001~0.002 mg·L^−1^ and 0.001~0.005 mg·L^−1^, respectively, which were all lower than the GBIII levels. Their CV were 9 ± 1%, 10 ± 1% and 13 ± 1%, respectively. While, Cd, Cr, and Zn in the river sediments were 0.08~0.12 mg·kg^−1^, 17~23 mg·kg^−1^ and 23~25 mg·kg^−1^, respectively, and all were lower than the B_i_. Their CV were 11 ± 1%, 13 ± 2%, and 15 ± 2%, respectively. These results indicated that Cd, Cr and Zn in the river water samples and sediments all were distributed uniformly and irregularly. In addition, since the water environment in the study area is mainly dominated by acidic and oxidizing conditions (Table 3) and the rock strata in the study area are dominated by carbonate, these conditions will actually accelerate the dissolution of carbonate and promote the release of PTEs [47,48], which is the main reason why the water environment in the study area is rich in Cd, Cr and Zn. Therefore, it infers that Cd, Cr and Zn were mainly from the natural environment [44].

### 3.2. Identification of Sources of PTEs

**Figure 4:**
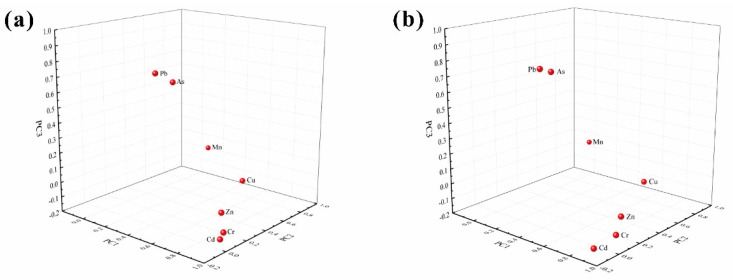
Factor analysis of PTEs in the river waters (**a**) and sediments (**b**).

The manganese mining and wasteyard are the main reasons for the serious PET pollution of river water and sediments in the research area. To further prove these observations, correlation matrix and multivariate statistical analyses were carried out. The Pearson’s correlation matrix and principal component analysis of PTEs in the water and sediments in the study area are shown in Table 4 and Table 5 and Figure 4.

In river water, the correlations among Cd, Cr and Zn were both significant (0.99 ≤ r ≤ 1, *p* < 0.01), indicating that these metals might originate from common sources [49]. Likewise, As and Pb showed significantly positive correlations (r = 0.99, *p* < 0.01) and weaker correlation with the elements of the first group (0.56 ≤ r ≤ 0.69, *p* < 0.05). At 0.05 significance level, the correlation coefficients of Cu with As, Cd, Cr, Pb, and Zn were 0.59, 0.64, 0.67, 0.61 and 0.69, respectively, and similar correlations also existed between Cu and Mn. These trends suggest that the Cu had many potential sources. In addition, three principal components (PCs) were obtained that explain 78.79% of the variation in the river water. PC1 was dominated by Cd, Cr, and Zn, accounting for 46.30% of the total variance. Cd was widely scattered in the lithosphere in the Karst area, and these three PTEs were at relatively low concentrations and low CV values. This trend indicates these elements mainly originated from nature [49]. Cu had a lower factor loading (0.57) than those of the other PTEs in PC1, whereas it had a higher factor loading of 0.75 in PC2. Similarly, As and Pb had relatively lower factor loadings of 0.47 and 0.48 in PC1, but they also had higher factor loadings in PC3. These showed that the three PTEs all had many sources [50]. PC2 explained 16.78% of the total variance and was dominated by Mn (0.98) and Cu (0.75). Generally, Mn is a common element in the lithosphere with a relatively high abundance and its concentration is not easily affected by artificial factors [9,50]. Despite being a common element in the lithosphere, the concentration and CV value of Mn is abnormally high in the study area, suggesting it mainly originated from the mining of manganese. Cu had a high concentration in K7 and a high CV value in the study area, but its concentrations were homogeneous in other sampling cross-sections. Cu was also always considered to be associated with Mn [22]. Therefore, Cu originated from both nature and manganese mining [51]. PC3 explained 15.71% of the total variance and was characterized with higher factor loadings of As (0.79) and Pb (0.83). These two PTEs both had a higher concentration in K16 and homogeneous concentration in other sampling cross-sections and they were typical Anthropogenic pollutants, suggesting they originated from both nature and the wasteyard [48,51].

In the case of river sediments, similar results were obtained. Cd, Cr and Zn showed strong positive correlation factors (0.98 ≤ r ≤ 0.99, *p* < 0.01), reinforcing the idea of a similar origin [51,52]. As and Pb showed similar significant positive correlations (r = 0.99, *p* < 0.01) and weaker correlation with the elements of Cd, Cr and Zn (0.63 ≤ r ≤ 0.67, *p* < 0.05). Cu also had some correlations with As, Cd, Cr, Pb, Zn and Mn (0.58 ≤ r ≤ 0.68, *p* < 0.05), indicating it had various sources in line with the river water. Similarly, three PCs were also obtained, explaining 83.23% of the variation in the river sediments. PC1 accounted for 48.89% of the variance and exhibited the higher loadings of Cd (0.97), Cr (0.99), Zn (0.95), as well as a moderate loading of As (0.48), Cu (0.53), Pb (0.50). PC2 and PC3 explained 17.67% and 16.67% of the total variance, respectively, and were dominated by Mn (0.945), Cu (0.71) and As (0.71), Pb (0.79). The results of the principal component analysis in the river sediments were consistent with those in the water samples in the study area, indicating that the sources of the river sediments were consistent with each other.

**Figure 5:**
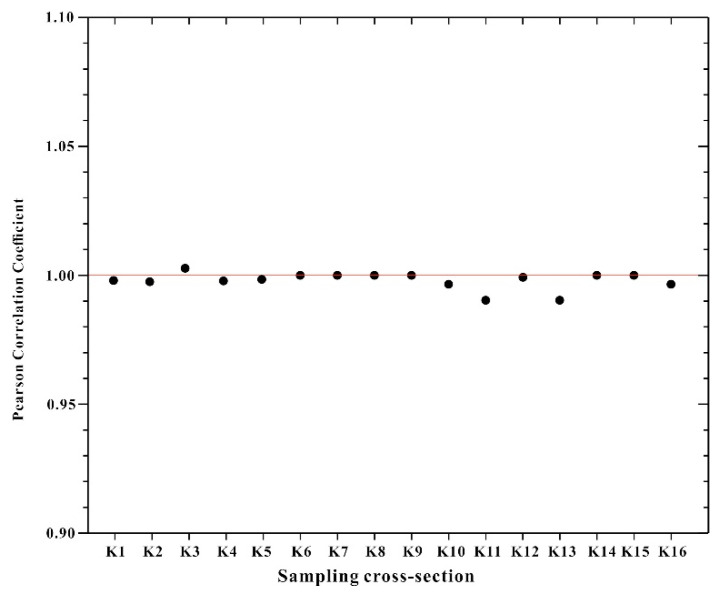
Pearson correlation coefficient of PTEs concentration in river waters vs. in sediments.

In order to validate the above analysis, the Pearson correlation coefficients between PET contents in the river water samples and sediments for those sampling cross-sections were showed in Figure 5. All of the sampling cross-sections exhibited a high relationship (r ≥ 0.99) between the PET concentration in both environmental compartments, indicating that they had common sources [12]. These are consistent with the above analysis, which further validates the accuracy of source analysis of PTEs in the river water and sediments.

### 3.3. Pollution Assessment of PTEs

**Figure 6:**
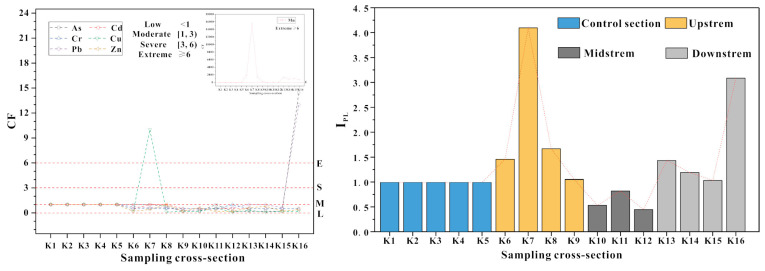
PTE pollution factors (contamination factor (CF) and I_PL_) in the river waters and sediments.

According to the contamination factor (CF) for the PTEs (As, Cd, Cr, Cu, Mn, Pb and Zn) in river water (Figure 6), Cu represented an extreme level at K7 in upstream, and As and Pb represented extreme level at K16 in downstream. Cu, As and Pb were low at other sites, while Mn showed extreme contamination at all the sites in the river. All sampling sections in the control area were at the critical contamination (CF = 1), which belonged to the low moderate contamination and they had a good contrast with the downstream.

Additionally, to further reflect the integrated influence of the PTEs in the river water of the study area, the I_PL_ of these seven PTEs are discussed (Figure 6). Results indicated that upstream and downstream were the main polluted sections of the Danshui River, which represented progressive contamination (I_PL_ > 1). Especially at K7 of upstream and K16 of downstream, it showed high I_PL_ values with 4 and 3. All sampling sites from midstream presented a level of non-pollution (I_PL_ < 1). The same as the CF, all sampling sections in the control section were also at the critical contamination (I_PL_ = 1), which belonged to the baseline pollution level, and they also had a good contrast with the downstream.

**Figure 7:**
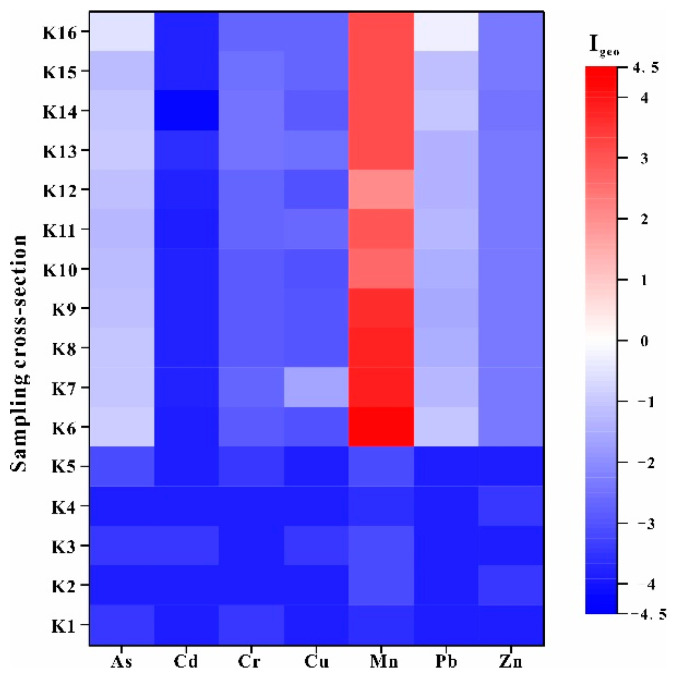
The I_geo_ distribution of PTEs from river sediments.

According to the Geo-accumulation Index (I_geo_) for the river sediments (Figure 7), only the Mn in the river sediments represented contamination (2 ≤ I_geo_ < 5), and the upstream and downstream are the main contaminated sections, which are heavily contaminated (3 ≤ I_geo_ < 4) and heavily to extremely contaminated (3 ≤ I_geo_ < 5), respectively. However, Mn in the river sediments in the midstream represented a moderate to heavy contamination (2 ≤ I_geo_ < 3), which suggested lower contamination_._ This was mainly due to the block by the dam at down part of upstream. Meanwhile, at other sites, there was lower contamination (I_geo_ < 0).

In order to determine the comprehensive influence from all PTEs, the I_PER_ was applied to evaluate the ecological risk from PTEs in river sediments (Table 6). Similar to the results of I_geo_, Mn upstream and downstream showed a level of moderate ecological risk and Mn was mainly related to the high concentration caused by manganese mining activities. The average ecological risk factors for the other PTEs were generally at low levels, which means the ecological risk in river sediments at acceptable levels.

According to these various indices (CF, I_PL_, I_geo_ and I_PER_), the main polluted sections by PTEs were upstream and downstream. PET pollutants were released from upstream and the sewage from upstream is discharged in the upper part of downstream after being utilized for hydropower. Ideally, these pollutants could be blocked by the dam at the lower part upstream. Mn was the main polluted PET. As and Pb in river water were at a relatively low pollution level downstream, and Cu was low upstream. The pollution levels of PTEs from river sediments were lower than in river water due to the PET pollutants are discharged mainly in the form of sewage, and the PET pollutants in river water are likely to precipitate into river sediments when the water environment changes. Therefore, the emission of sewage is the main pollution source of the study area and water conservancy facilities were the key impacts to the pollution distribution of PTEs. Therefore, the control of sewage emission should be strengthened and the water conservancy facilities should be utilized later on for additional pollution control. These are of great significance to the continued pollution control of the river in the mining areas.

## 4. Conclusions

In the study, the source, pollution distribution and potential ecological risk of PTEs (As, Cd, Cr, Cu, Mn, Pb and Zn) in the river water samples and sediments of the Gucheng manganese mining area were analyzed. This study provides a valuable reference for other mining areas that have similar river water and sediments pollution levels and can assist with the control and remediation of PET pollution. The conclusions based on this study are as follows:

(1) PET pollution from manganese mining in the Danshui River was analyzed and it was determined that the main pollutant was Mn, which exhibited concentrations higher than the GBIII and C_BG_ levels. The river water environment was mainly acidic and oxidizing and only the spatial distribution of Mn was greatly affected by the water environment. The pollution distribution of Mn was dominated by natural processes and anthropogenic activities. Upstream and downstream of the river were the main polluted areas, while midstream was lower. The water environment in the study area had a significant influence on the state of Mn as well as its pollution distribution. In addition, the concentration of As and Pb in the river water and sediments were high at K16, and Cu was high at K7. All of these locations were located upstream and downstream of the river.
(2) Manganese mining activities were the main source of pollution. A wasteyard located along the river was also identified as a source of pollution. According to Pearson’s correlation and PCA, it showed that three groups of PTEs, Cd, Cr and Zn, mainly originated from nature and Pb and As were mainly related to the wasteyard. Mn mainly originated from manganese mining activities, and Cu was from both manganese mining activities and the wasteyard.
(3) According to the CF and I_PL_, As and Pb were low in river water from downstream, and Cu in river water was higher upstream. Mn was considered as the main pollutant of the river water in the whole river. Upstream and downstream were the main polluted sections of river water. Based on the results of I_geo_ and I_PER_, river sediments were heavily polluted by Mn and the other PTEs were at normal levels in this area. The I_PER_ of PTEs in the river sediments were mostly at a low-risk level, only Mn in the upstream showed a moderate ecological risk, downstream showed low ecological risk and higher risk than midstream. Therefore, Mn was the main pollutant in the upstream and downstream polluted sections of the river.
(4) According to the pollution distribution of PTEs, element speciation, the discharge of pollutants and water conservancy facilities were the key impacts observed and should be considered for the pollution treatment and environmental protection in this study area.

## Figures and Tables

**Table 1:** The classification criteria for potential ecological risk index.

Classification	$E_{r}^{i}$		RI	
1	<16	Low Risk	<60	Low Risk
2	(16,32]	Moderate Risk	(60,120]	Moderate Risk
3	(32,64]	Considerable Risk	(120,240]	Considerable Risk
4	(64,128]	High Risk	(240,480]	High Risk
5	>128	Very High Risk	>480	Very High Risk

**Table 2:** Coefficient of variation and standard deviation (CV ± SD%) of potentially toxic elements (PTEs) in Danshui River.

Sampling Size	Element	As	Cd	Cr	Cu	Mn	Pb	Zn
48	CV in river sediments (Mean ± SD%)	35 ± 2	11 ± 1	13 ± 2	55 ± 4	206 ± 12	37 ± 2	15 ± 2
48	CV in river water (Mean ± SD%)	36 ± 3	9 ± 1	10 ± 1	36 ± 3	103 ± 2	35 ± 2	13 ± 1

SD: standard deviation.

**Table 3:** pH and Eh of PTEs in river water.

Sampling Cross-Section	Sampling Size	pH (Mean ± SD)	Eh/mV (Mean ± SD)
K1	3	8.56 ± 0.1	290.6 ± 2.1
K2	3	8.58 ± 0.2	310.5 ± 2.2
K3	3	8.69 ± 0.2	312.2 ± 2.6
K4	3	8.88 ± 0.1	324.5 ± 2.3
K5	3	8.79 ± 0.2	299.8 ± 2.5
K6	3	6.01 ± 0.1	300.8 ± 1.9
K7	3	6.08 ± 0.2	300.4 ± 1.8
K8	3	6.18 ± 0.2	300.8 ± 1.5
K9	3	6.98 ± 0.2	301.9 ± 2.1
K10	3	8.11 ± 0.1	335.6 ± 2.2
K11	3	7.98 ± 0.2	301.9 ± 1.2
K12	3	8.45 ± 0.2	330.8 ± 1.4
K13	3	6.14 ± 0.1	300.9 ± 1.6
K14	3	6.26 ± 0.2	302.2 ± 1.9
K15	3	6.26 ± 0.1	301.9 ± 1.1
K16	3	6.78 ± 0.1	310.5 ± 2.0

SD: standard deviation.

**Table 4:** Correlation matrix of PTEs in water and sediment of the river sections in manganese mining areas.

Element in Water	As	Cd	Cr	Cu	Mn	Pb	Zn
As	1	0.65 *	0.69 *	0.59 *	0.16	0.99 **	0.69 *
Cd		1	0.98 **	0.64 *	0.26	0.69 *	0.99 **
Cr			1	0.67 *	0.30	0.66 *	0.99 **
Cu				1	0.66 *	0.61 *	0.69 *
Mn					1	0.26	0.33
Pb						1	0.56 *
Zn							1
**Element in Sediment**	As	Cd	Cr	Cu	Mn	Pb	Zn
As	1	0.63 *	0.67 **	0.58 *	0.15	0.99 **	0.67 *
Cd		1	0.98 **	0.62 *	0.24	0.66 *	0.99 **
Cr			1	0.66 *	0.31	0.66 *	0.99 **
Cu				1	0.63 *	0.62 *	0.68 *
Mn					1	0.21	0.34
Pb						1	0.56 *
Zn							1

** and * indicate that the correlation is significant at the 0.01 and 0.05 level, respectively.

**Table 5:** Principal component analysis of PTEs in water and sediment of Danshui. (The bold numbers in the table indicate they have higher factor scores).

Element in Water	PC1	PC2	PC3	Element in Sediment	PC1	PC2	PC3
As	**0.47**	0.01	**0.79**	As	**0.48**	0.13	**0.71**
Cd	**0.94**	−0.16	−0.13	Cd	**0.97**	−0.02	−0.13
Cr	**0.99**	−0.02	−0.08	Cr	**0.99**	−0.01	−0.08
Cu	**0.57**	**0.75**	0.04	Cu	**0.53**	**0.71**	0.04
Mn	−0.04	**0.98**	0.06	Mn	0.10	**0.95**	0.02
Pb	**0.48**	−0.09	**0.83**	Pb	**0.50**	−0.04	**0.79**
Zn	**0.98**	0.04	0.06	Zn	**0.95**	0.02	0.02
eigenvalues	3.13	1.12	1.11	eigenvalues	3.16	1.27	1.11
variance/%	46.30	16.78	15.71	variance/%	48.89	17.67	16.67
cumulative variance/%	46.30	63.08	78.79	cumulative variance/%	48.89	66.56	83.23

**Table 6:** Potential ecological risk posed by individual PTE (E_i_) and multiple PTEs (RI) in the river sediments.

River Section	Sampling Size	E_i_ (Mean ± SD)							RI (Mean ± SD)
		As	Cd	Cr	Cu	Mn	Pb	Zn	
Upstream	12	7.4 ± 0.4	3.2 ± 0.1	0.4 ± 0.0	1.3 ± 0.6	22.4 ± 4.1	3.0 ± 0.4	0.3 ± 0.0	38.1 ± 4.8
Midstream	9	6.6 ± 0.3	3.2 ± 0.1	0.4 ± 0.0	1.0 ± 0.1	9.0 ± 2.2	2.8 ± 0.1	0.3 ± 0.0	23.4 ± 1.9
Downstream	12	7.9 ± 1.5	3.2 ± 0.4	0.5 ± 0.0	1.2 ± 0.1	13.1 ± 0.2	4.0 ± 1.2	0.3 ± 0.0	30.1 ± 2.7

SD: standard deviation.

## Data Availability

All relevant data sets in this study are described in the manuscript.
